# Doxorubicin-Incorporated Nanoparticles Composed of Ce6-Conjugated Hyaluronic Acid-*b*-poly(ethylene glycol) Copolymer for Overcoming Doxorubicin Resistance of Breast Cancer Cells

**DOI:** 10.3390/ijms27156993

**Published:** 2026-08-04

**Authors:** Tae Hyeon Kim, Kyung-Jin Oh, Myeong Yoo Park, Ilkeun Kong, Jaewon Jo, Young-Ju Lee, Hyo-Young Lee, Doug-Hoon Kim, Jinsu Park, Jae-Woon Nah, Young-IL Jeong

**Affiliations:** 1 Division of Animal Science, Gyeongsang National University, Jinju 52828, Republic of Korea; tea9330@naver.com (T.H.K.); ikong7900@gmail.com (I.K.); 2 Department of Urology, Chonnam National University Hospital, Chonnam National University Medical School, Gwangju 61469, Republic of Korea; okj1225@jnu.ac.kr (K.-J.O.); gkrauddy1@naver.com (M.Y.P.); 3 Gwangju Center, Korea Basic Science Institute, Gwangju 61186, Republic of Korea; jawon1119@kbsi.re.kr (J.J.); yjlee@kbsi.re.kr (Y.-J.L.); 4 Department of Radiological Science, Dong-Eui University, Pusan 47340, Republic of Korea; lhy250@deu.ac.kr; 5 Department of Optometry, Masan University, Changwon 51217, Republic of Korea; doukhoon@naver.com; 6 The Korean Association for the Industrial Technology Security, Seoul 06732, Republic of Korea; inversion@kaits.or.kr; 7 Kittolife, Pyeongtaek-si 17749, Republic of Korea; jwnah@hanmail.net; 8 Institute of Well-Aging Medicare & Chosun University G-LAMP Project Group, Chosun University, Gwangju 61452, Republic of Korea

**Keywords:** drug-resistant cancer, nanoparticles, chemotherapy, photodynamic therapy, reactive oxygen species, tumor targeting

## Abstract

Oxidative stress in the tumor microenvironment, which is its own intrinsic property, is frequently utilized to deal with the drug-targeting issue in the nanoparticle drug delivery system. For this purpose, reactive oxygen species (ROS)-sensitive nanoparticles encapsulating doxorubicin (DOX) and chlorin e6 (Ce6) were synthesized for treatment of MDA-MB-231 breast cancer cells. Hyaluronic acid (HA) with a reductive end was conjugated with methoxy poly(ethylene glycol) (PEG) using thioketal diamine (ThdNH_2_) linkage (HA-*b*-PEG copolymer). Then, Ce6 were conjugated to the carboxylic acid group of HA via ThdNH_2_ (HA(Ce6)-*b*-PEG copolymer). DOX was physically incorporated to make DOX-incorporated HA(Ce6)-*b*-PEG copolymer nanoparticles (DOX-NP). HA(Ce6)-*b*-PEG copolymer nanoparticles (empty NP) and DOX-NP have a tiny particle size, less than 200 nm, with spherical morphology. They were responsively disintegrated according to the hydrogen peroxide (H_2_O_2_) concentration, then the release rate of Ce6 or DOX was accelerated, indicating that empty NP and DOX-NP have ROS sensitivity. DOX-resistant MDA-MB-231 cells were prepared by continuous treatment of DOX for three months. DOX-NP were efficiently internalized into the cells while intra-cellular delivery of DOX itself was inhibited. DOX-NP has higher anticancer activity against DOX-resistant MDA-MB-231 cells than that of DOX itself since cells were resistant to DOX itself. Under light irradiation, DOX-NP significantly decreased the viability of DOX-resistant MDA-MB-231 cells while DOX itself did not properly affect cell viability. Empty NP also efficiently inhibited cell viability rather than that of Ce6 itself while both of them did not affect the cell viability in the absence of light irradiation. Furthermore, empty NP showed higher ROS generation than that of Ce6 itself. DOX-NP more efficiently induced apoptosis/necrosis than DOX itself. In DOX-resistant MDA-MB-231 cell-bearing mice, DOX-NP was efficiently delivered to tumor tissue. DOX-NP greatly inhibited the growth of tumors under light irradiation, more than that of DOX itself or empty NP. In conclusion, DOX-NP showed promising antitumor activity against DOX-resistant MDA-MB-231 cells.

## 1. Introduction

Nano-scaled vehicles have been extensively investigated in the field of biomedical and drug delivery systems [1,2,3]. Among various nanomaterials and nano-scaled vehicles, nanoparticles for drug delivery systems have intrinsic properties such as huge surface area, small particle sizes, targetability, controlled drug release, versatile administration and overcoming biological barriers [3,4,5]. Since tumor tissues have leaky, dysfunctional blood vessels and poor lymphatic drainage, nanoparticles are promising candidates for tumor targeting via enhanced permeability and the retention effect of nanoparticles [5,6]. Physiological properties of the tumor microenvironment have unique properties such as overexpression of various receptors, acidic pH, elevated reduction/oxidation (redox) reaction and enhanced metabolism [7]. These different characteristics of tumor vs. normal tissues enable tumor-specific targeting for nanoparticles, i.e., nanoparticles can be designed to be sensitive to acidic pH and/or elevated redox potential, and then cytotoxic anticancer agents can be specifically liberated in the tumor tissues [8,9]. For example, Lim et al. reported that pH-sensitive nanoparticles selectively liberated the JNK inhibitor, sensitized brain cancer to radiation treatment and then efficiently suppressed the growth of brain tumors [10]. Furthermore, acidic pH and reactive oxygen species (ROS)-dual-sensitive nanoparticles suppressed pulmonary metastatic potential of colon cancer cells through enhanced drug release properties at acidic pH and oxidative stress [11].

Chemotherapy still remains one of the major treatment options for cancer. It is known that anticancer agent-based chemotherapy has reasonable effectiveness in reducing tumor size and eradicating microscopic cancer cells [12]. It facilitates success rates of subsequent treatment options such as surgeries, radiation therapy, and immunotherapies [12]. Various chemotherapeutic agents have been tried to treat cancer patients. Among them, doxorubicin (DOX) is one of the most popular chemotherapeutic agents for malignant lymphoma, leukemia, and soft tissue sarcoma [13]. The basic anticancer effect of DOX interferes with the action of DNA topoisomerase II and then induces damage of the DNA of tumor cells [13]. These actions of DOX induce the inhibition of the tumor cell proliferation and then reduce the tumor size [13]. In the clinical application, serious side effects of DOX have been reported such as hair loss, stomatitis, nausea, vomiting, tissue necrosis and suppression of bone marrow function [14]. Furthermore, cardiotoxicity, hepatotoxicity, nephrotoxicity and/or neurotoxicity seriously inhibit the DOX-based chemotherapeutic approach for cancer patients [15]. The drug-resistant issues of DOX frequently discourage therapeutic processes and results [16,17,18]. To solve these problems, various strategies have been tried [16,17,18,19,20,21]. For example, intravenous infusions of ifosfamide/mesna reduced DOX-resistant problems by depletion of intracellular glutathione (GSH), since elevated intracellular GSH levels are seriously associated with DOX resistance [16]. Smoots et al. reported that the histone deacetylase (HDAC) inhibitor accelerates apoptotic death of DOX-resistant triple-negative breast cancer cells and then inhibits tumor growth in vivo [18]. Interestingly, nanoparticle-based drug delivery systems are reported as a promising candidate for overcoming drug resistance problems [21]. Nanoparticle-based drug delivery systems have the potential to deliver anticancer drugs intracellularly and then alleviate DOX resistance through alteration of the specific molecular mechanisms of multi-drug resistance such as drug efflux transporter overexpression, aberrations of topoisomerase IIα, apoptosis signal impairments, oncogenic activation, tumor heterogeneity and cancer stem cells [22]. Nanoparticle-based drug delivery systems can be applicable in overcoming DOX resistance.

Photodynamic therapy (PDT) is considered a promising candidate for the treatment of cancer because PDT is composed of light, oxygen and photosensitizers [23]. Since photosensitizers are only activated then kill the cancer cells by producing reactive oxygen species (ROS), PDT is a safe treatment modality for cancer therapy. In other words, photosensitizers have negligible toxicity against normal cells/tissues in the absence of light irradiation [23,24]. Photosensitizers are also used as a diagnostic tool for the detection of cancer because photosensitizers are fluorescently activated under light irradiation with specific wavelengths [25,26]. For example, Cui et al. reported that chlorin e6 (Ce6)-decorated nanoparticles can be applied to diagnose the progression of breast cancer since breast cancer is closely associated with hypoxia [25]. Then, they argued that Ce6-decorated nanoparticles can be used to monitor therapeutic processes using oxygen-generating nanoparticles. Since the resection quality of brain tumors affects the recurrence-free survival in patients having glioblastoma, photosensitizers such as 5-aminolevulinic acid and Ce6 can be used to detect the resection field and then to resect fluorescent tissues micro-surgically [26]. Furthermore, a photoactivable codelivery system using photosensitizers achieved a high encapsulation capacity of paclitaxel and efficiently inhibited the growth of cancer cells in vitro/in vivo through a dual strategy of PDT-chemotherapy [27]. PDT together with chemotherapeutic agents, especially, efficiently inhibits the growth of cancer cells [28,29]. Fang et al. reported that redox-responsive polymeric micelles encapsulated with paclitaxel and indocyanine green efficiently induce apoptosis/necrosis of tumor cells [28]. Photosensitizer-based PDT combined with anticancer agents especially resulted in intracellular delivery of anticancer drugs and then induced improved anticancer activity against DOX-resistant breast cancer cells [30]. Canti et al. reported that PDT in the presence of DOX has promising anticancer activity both of DOX-resistant and -sensitive cells while DOX has low anticancer activity against DOX-resistant cell lines [31]. Furthermore, Yang et al. also reported that nanostructures composed of Rose Bengal-conjugated human serum albumin and DOX-loaded CaCO_3_ can be delivered intracellularly against DOX-resistant cancer cells [32]. They argued that their nanostructures are promising candidates for drug-resistant problems. Their nanostructures showed promising anticancer activity at low-dose PDT.

In this study, we synthesized the Ce6-conjugated hyaluronic acid-*b*-methoxy poly(ethylene glycol) (HA(Ce6)-*b*-PEG) copolymer via a thioketal linker and then an anticancer drug, DOX, was incorporated for improved anticancer treatment against DOX-resistant MDA-MB-231 breast cancer cells. Physicochemical properties of nanoparticles were investigated and the *in vitro*/*in vivo* anticancer activity of them was investigated using MDA-MB-231 breast cancer cells.

## 2. Results

### 2.1. Synthesis and Characterization of HA(Ce6)-b-PEG Copolymer

**Figure 1:**
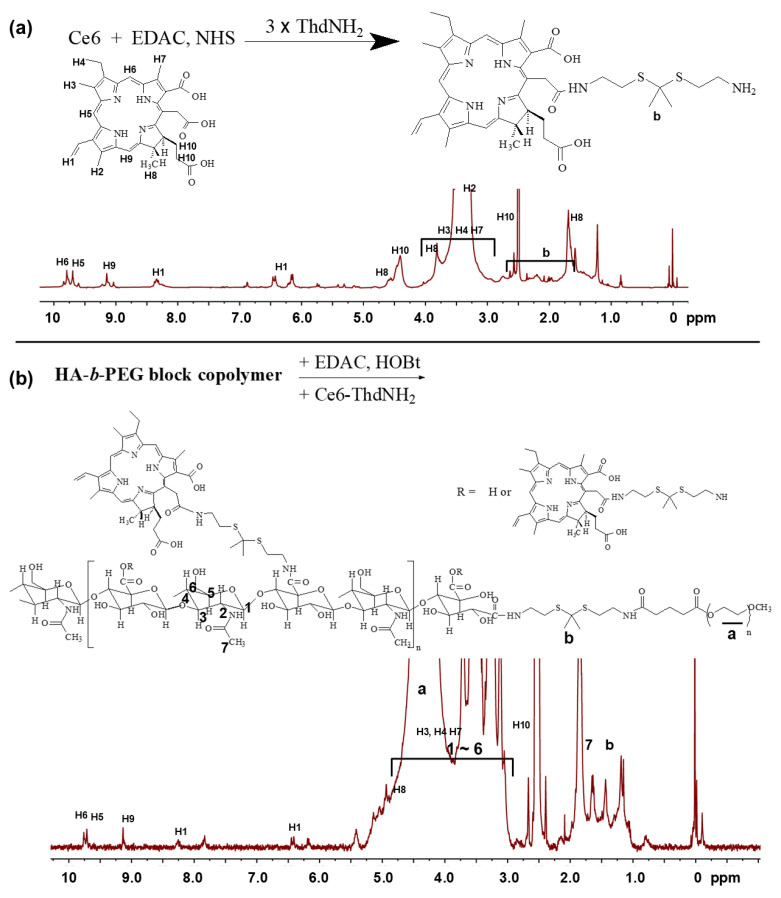
Synthesis scheme and ^1^H NMR spectra Ce6-ThdNH2 conjugates (**a**) and HA(Ce6)-*b*-PEG copolymer (**b**).

To synthesize the HA(Ce6)-*b*-PEG copolymer, methoxy poly(ethylene glycol) (PEG) was reacted with an excess amount of thioketal diamine (ThdNH_2_) (PEG-ThdNH_2_) to make PEG-ThdNH_2_, as shown in Figure S1a. As shown in Figure S1b, specific peaks of PEG and ThdNH_2_ were observed from ^1^H nuclear magnetic resonance (NMR) spectra. HA with a reductive end was prepared by treatment of NaBH_3_CN and then conjugated with PEG-ThdNH_2_ to make the HA-*b*-PEG copolymer, as shown in Figure S2a,b. Specific peaks of HA, PEG and ThdNH_2_ were observed as shown in Figure S2b. To conjugate chlorin e6 (Ce6), Ce6 was conjugated with ThdNH_2_ to make ROS-sensitive conjugates as shown in Figure 1a. Specific peaks of Ce6 and ThdNH_2_ at ^1^H NMR spectra were confirmed at ^1^H NMR spectra between 1 and 10 ppm as shown in Figure 1a. After that, Ce6-ThdNH_2_ conjugates were conjugated with carboxyl acid of the HA-*b*-PEG copolymer as shown in Figure 1b. Specific peaks of Ce6, HA and PEG were observed with ^1^H NMR spectra, indicating that the Ce6-conjugated HA-*b*-PEG copolymer (HA(Ce6)-*b*-PEG copolymer) was synthesized. Ce6 contents in the HA(Ce6)-*b*-PEG copolymer were 5.4% (*w*/*w*).

In FT-IR spectra (Figure S3a–d), specific peaks of Ce6 were shown between 600 and 1800 cm^−1^, i.e., the bands around 1710 cm^−1^ and 1597 cm^−1^ were assigned to the C-O stretch and C=O stretch of the -COOH group. In addition, the bands between 1300 and 1500 cm^−1^ corresponded to the fingerprint region as shown in Figure S3a. Specific peaks of HA were shown between 900 and 3800 cm^−1^, i.e., vibrations of OH stretching and NH stretching in the N-acetyl side chain were observed at 3348 cm^−1^ (Figure S3b). The two bands at 2926 and 2927 cm^−1^ were confirmed to be glucuronic acid due to the symmetric methyl C-H stretching. The peaks at 1637 and 1421 cm^−1^ were confirmed to be the amide I group of C=O carboxyl and primary aromatic amine of C-N stretching. The 1023 cm^−1^ were confirmed to alcohol C-O stretching. In PEG peaks (Figure S3c), the peaks in 1240 cm^−1^ and 1100 cm^−1^ were associated with C-O (alcohol) and C-O-C, respectively. The spectrum between 1320 and 1380 cm^−1^ was associated with the CH_2_ wagging region. Figure S3d showed the synthesized HA(Ce6)-*b*-PEG copolymer.

### 2.2. Characterization of DOX-Incorporated HA(Ce6)-b-PEG Nanoparticles (DOX NP)

**Figure 2:**
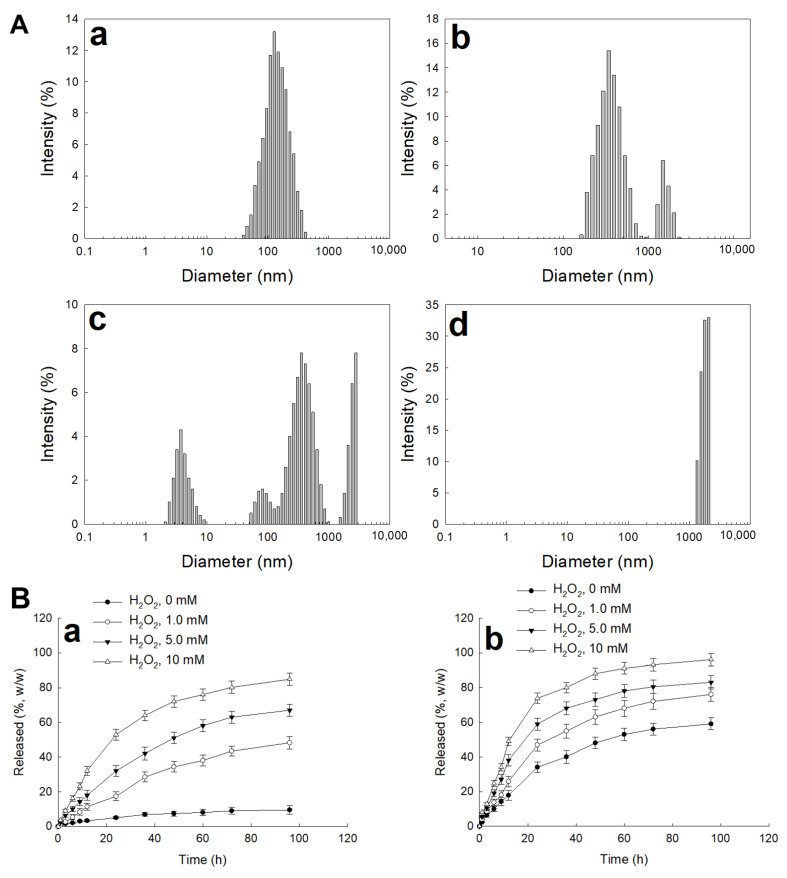
Characterization of DOX-incorporated HA(Ce6)-*b*-PEG copolymer nanoparticles. (**A**). Changes in particle size distribution according to H_2_O_2_ concentration. (**a**) H_2_O_2_, 0 mM; (**b**) H_2_O_2_, 1 mM; (**c**) H_2_O_2_, 5 mM; (**d**) H_2_O_2_, 10 mM. (**B**). The effect of oxidative stress on the drug release properties. (**a**) Ce6 release from HA(Ce6)-*b*-PEG copolymer nanoparticles (empty NP); (**b**) DOX release from DOX-incorporated HA(Ce6)-*b*-PEG copolymer nanoparticles (DOX-NP).

To make DOX-incorporated nanoparticles, the HA(Ce6)-*b*-PEG copolymer was dissolved in DMSO/H_2_O mixtures and then mixed with DOX. Since DOX itself has a cationic property and the HA(Ce6) part in the HA(Ce6)-*b*-PEG copolymer has an anionic property, DOX can be incorporated into the nanoparticles through ionic interaction and hydrophobic interaction between DOX and the HA(Ce6) part. Intrinsic properties of DOX-incorporated HA(Ce6)-*b*-PEG copolymer nanoparticles (DOX-NP) are summarized in Table 1. As shown in Table 1, experimental DOX contents were 8.9% (*w*/*w*) and they have small particle sizes less than 200 nm. Furthermore, colloidal stability of empty NP and DOX NP was measured for one week as shown in Figure S4. Even though average particle sizes of empty NP and DOX NP were slightly increased, their average particle sizes were not significantly changed for one week, indicating that empty NP and DOX NP were stable in aqueous condition. Nanoparticles of the HA(Ce6)-*b*-PEG copolymer (empty NP) have a smaller diameter than DOX NP, indicating that diameter was increased by DOX incorporation. Furthermore, DOX NP showed monomodal particles distributed in miniscule sizes, as shown in Figure 2A(a). To investigate ROS sensitivity, DOX-NP was incubated in the presence of H_2_O_2_ (1.0 mM (Figure 2A(b)), 5.0 mM (Figure 2A(c)) and 10 mM (Figure 2A(d)) as shown in Figure 2A,B. When H_2_O_2_ was added to aqueous solution of nanoparticles, size distribution was changed from monomodal to multimodal distribution patterns as shown in Figure 2A. Practically, particle size measurement failed at 10 mM H_2_O_2_. As shown in Figure 2B(a), Ce6 release rate was accelerated under the presence of H_2_O_2_ and then release rate became increased according to the concentration of H_2_O_2_ while Ce6 release rate was significantly lower in the absence of H_2_O_2_. Furthermore, DOX release rate also accelerated under the presence of H_2_O_2_ (Figure 2B(b)). These results indicated that HA(Ce6)-*b*-PEG copolymer nanoparticles (empty NP) have ROS sensitivity and they have oxidative-specific delivery capacity.

### 2.3. In Vitro Cell Culture Study

**Figure 3:**
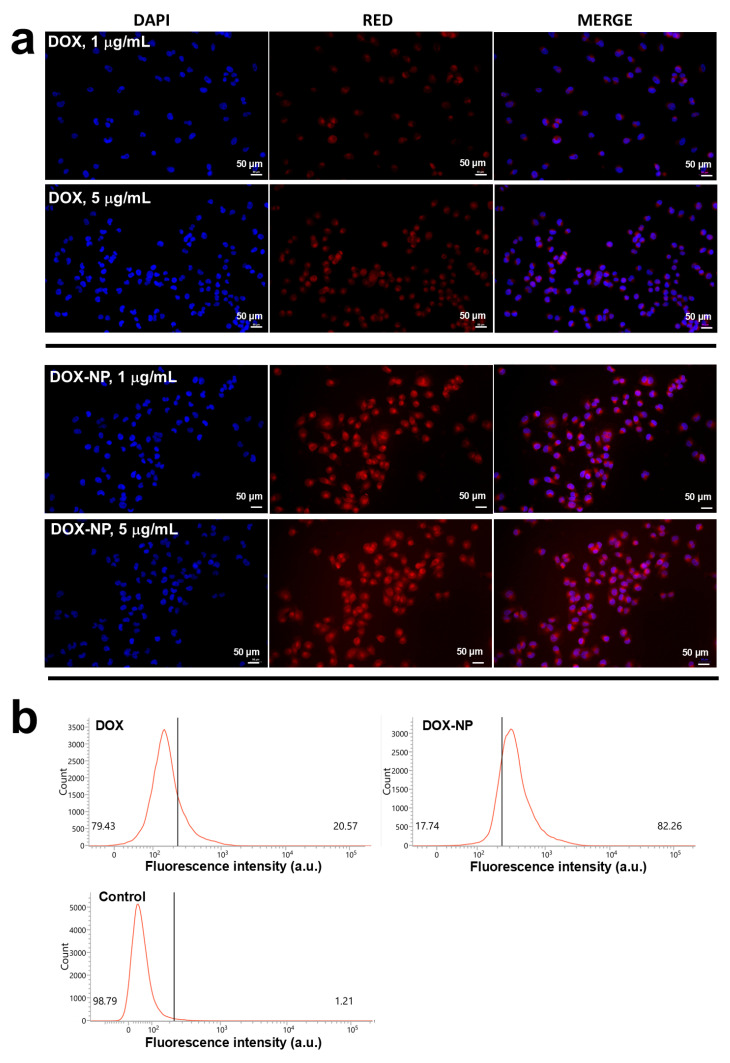
Intracellular delivery of DOX or DOX-NP against MDA-MB-231 cells. (**a**) Fluorescence microscopic images; (**b**) Flow cytometric analysis. DOX or DOX-NP was treated to MDA-MB-231 cells for 2 h. Then, cells were observed with fluorescence microscope or analyzed with flow cytometer. Scale bar, 100 µm.

To study anticancer activity, MDA-MB-231 cells were continuously treated with 0.001 μg/mL DOX and then DOX concentration was gradually increased for three months to make DOX-resistant MDA-MB-231 cells (Figure S5). DOX-resistant MDA-MB-231 cells showed relatively higher cell viability compared to MDA-MB-231 cells themselves (Figure S5a). Furthermore, IC_50_ values of DOX-resistant MDA-MB-231 cells were significantly higher than MDA-MB-231 cells (Figure S5b). As shown in Figure 3a,b, the red fluorescence of DOX-resistant MDA-MB-231 cells was higher at DOX-NP treatment rather than that of DOX itself, indicating that DOX-resistant MDA-MB-231 cells inhibited intracellular delivery of DOX itself (Figure 3a) while DOX-NP was efficiently delivered to the intracellular region of DOX-resistant MDA-MB-231 cells. Flowcytometric analysis also shows similar results, i.e., the intensity of DOX-NP treatment was higher than the intensity of DOX itself (Figure 3b).

**Figure 4:**
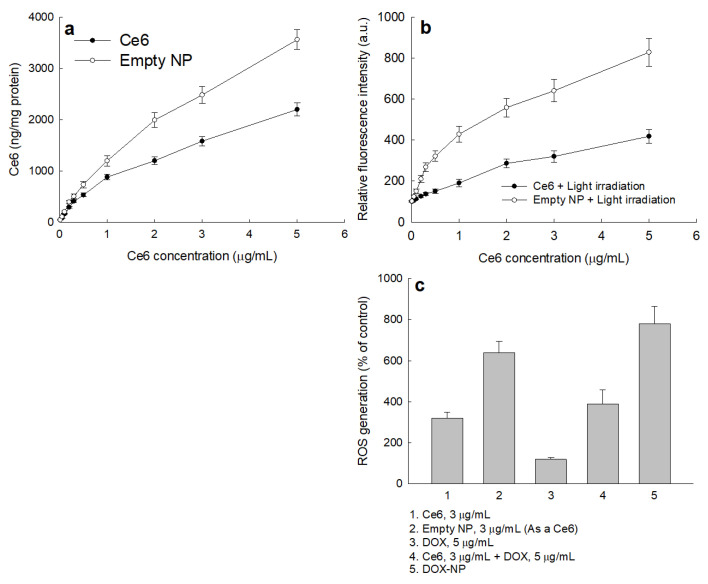
The effect of Ce6 or empty NP on the Ce6 uptake and ROS generation at DOX-resistant MDA-MB0231 cells. (**a**) Ce6 uptake; Ce6 vs. empty NP, *p* < 0.001. (**b**) ROS generation; Ce6 + light irradiation vs. empty NP + light irradiation, *p* < 0.001. (**c**) Ce6, empty NP (HA(Ce6)-*b*-PEG copolymer), DOX, DOX + Ce6 and DOX-NP on the generation of intracellular ROS.

Figure 4 showed the PDT effect of Ce6, DOX, empty NP and/or DOX NP. As shown in Figure 4a,b, the Ce6 uptake ratio and ROS generation of Ce6 and empty NP were dose-dependently increased. Empty NP especially showed a higher Ce6 uptake ratio and ROS generation than that of Ce6 itself. When DOX + Ce6 or DOX NP was treated to cells, the intracellular ROS level was higher than those of Ce6 itself or empty NP (Figure 4c). To investigate whether or not DOX itself affects the generation of DOX, DOX itself was treated to cells and then irradiated as shown in Figure S6. Even though DOX itself slightly increased intracellular ROS generation, ROS generation by DOX itself was practically negligible by light irradiation (Figure S6).

**Figure 5:**
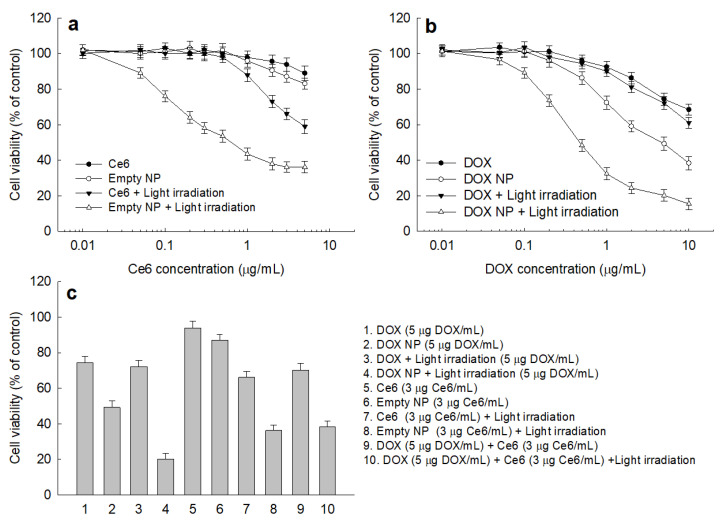
Cell viability of Ce6, empty NP, DOX and/or DOX-NP-treated DOX-resistant MDA-MB-231 cells. (**a**). The effect of Ce6 and empty NP in the absence and presence of light irradiation. Ce6 vs. empty NP, *p* < 0.01. (**b**). The effect of DOX and DOX-NP in the absence and presence of light irradiation. DOX vs. DOX-NP, *p* < 0.001. (**c**). Comparison of DOX, DOX NP, Ce6 and empty NP against DOX-resistant MDA-MB-231 cells. Ce6 and DOX concentration was adjusted to 3 μg/mL and 5 μg/mL based on drug contents of empty NP and DOX NP. Cells were exposed to DOX, DOX NP, Ce6, empty NP or Ce6 + DOX combination and, after 2 h, irradiated at 664 nm (The dose of light: 2.0 J/cm^2^). Cells were further incubated for 22 h in CO_2_ (37 °C).

Figure 5 shows the effect of DOX, Ce6, empty NP and DOX-NP on the cell viability of DOX-resistant MDA-MB-231 cells. Under the dark condition (Figure 5a), cell viability was not significantly decreased until 5 μg/mL Ce6 concentration in the absence of light irradiation, indicating that both Ce6 and empty NP have no intrinsic cytotoxicity against DOX-resistant MDA-MB-231 cells (Figure 5a). When cells were irradiated, the viability of DOX-resistant MDA-MB-231 cells dose-dependently decreased according to the concentration of Ce6, both of Ce6 itself and empty NP. Otherwise, DOX itself did not affect the cell viability by light irradiation, i.e., cell viability was not significantly changed by light irradiation when DOX itself was treated (Figure 5b). DOX-NP showed dose-dependent cytotoxicity against DOX-resistant MDA-MB-231 cells, i.e., cell viability was significantly decreased by DOX NP in the presence of light irradiation compared to the absence of light irradiation, indicating that DOX-based chemotherapy and Ce6-based PDT have a promising anticancer effect against DOX-resistant MDA-MB-231 cells. These results indicated that DOX-NP has the potential to produce ROS and then induce apoptosis/necrosis of cancer cells. As shown in Table 2, the IC_50_ value of empty NP and/or DOX-NP was significantly lower than that of Ce6 or DOX, indicating that empty NP and DOX NP have superior anticancer activity and have the potential to overcome the DOX-resistant problem of MDA-MB-231 cells. The IC_50_ value of DOX NP under light irradiation was estimated as 0.44 µg/mL and the practical Ce6 content in the DOX NP was calculated as 0.24 µg/mL. The cell viability of empty NP at 0.2 µg/mL and 0.3 µg/mL Ce6 concentration under light irradiation was 64.05 % and 58.06 % respectively. These results can be compared to DOX NP (5 µg/mL DOX) in the absence of light irradiation, which resulted in 50% cell viability.

**Figure 6:**
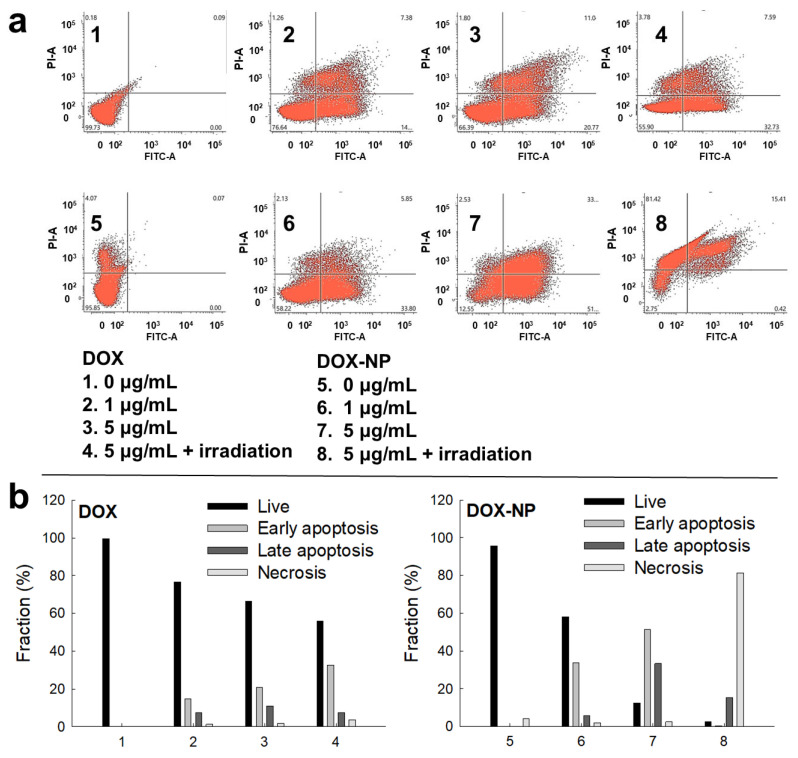
(**a**) Apoptosis/necrosis analysis of DOX-resistant MDA-MB-231 cells. DOX or DOX-NP were treated to DOX-resistant MDA-MB-231 cells. After 2 h, cells were irradiated at 664 nm (2 J/cm^2^) and then further incubated for 22 h in CO_2_ (37 °C) and then apoptosis/necrosis of cells was analyzed. (**b**) Apoptosis and/or necrosis fraction of cells abbreviated from panel (**a**).

Since NP showed higher ROS generation against DOX-resistant MDA-MB-231 cells than that of Ce6 itself, DOX-NP efficiently induced apoptosis/necrosis of DOX-resistant MDA-MB-231 cells (Figure 6a,b). In other words, apoptosis/necrosis of DOX-resistant MDA-MB-231 cells increased by treatment both of DOX or DOX-NP. Necrosis significantly increased by treatment of DOX-NP under light irradiation. These results indicate that DOX-NP has superior anticancer activity against DOX-resistant MDA-MB-231 cells.

### 2.4. Animal Tumor Imaging and Antitumor Activity Using Tumor Xenograft Model

**Figure 7:**
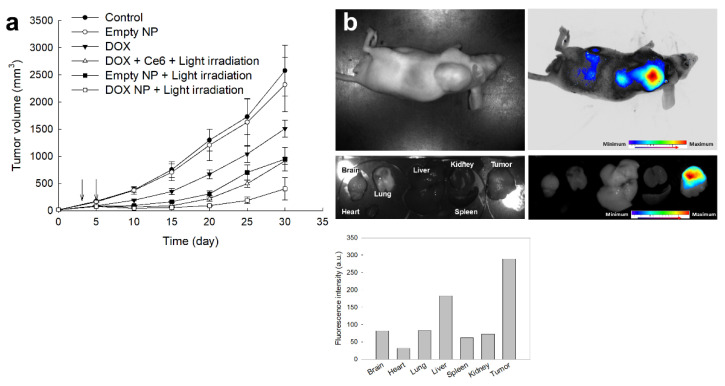
(**a**) Antitumor effect of DOX and DOX-NP on the MDA-MB-231-bearing mice. For antitumor activity, DOX, empty NP, DOX + Ce6 or DOX-NP were i.v. administered via tail vein of mice. Three days and five days later, mice that were administered with DOX + Ce6, empty NP or DOX-NP were irradiated with light at 664 nm (5.0 J/cm^2^). Each group was composed of five mice (*n* = 5) and expressed as average ± SD. DOX vs. DOX-NP + light irradiation, *p* < 0.01; empty NP vs. empty NP + light irradiation, *p* < 0.01. (**b**) Animal fluorescence imaging. DOX-NP (10 mg/kg as a DOX) was intravenously (i.v.) administered via tail vein of mice and, 24 h later, mice were observed with imaging equipment.

To study the antitumor activity of DOX-NP, DOX-resistant MDA-MB-231 cells were implanted into the back of mice. Three weeks later, PBS, DOX, empty NP, DOX + Ce6 or DOX-NP were intravenously (i.v.) administered via the tail vein of mice for antitumor activity (Figure 7a). Tumor volume gradually increased according to the time course. When animals were treated with DOX, the growth of tumor volume decreased compared to the control group. Practically, NP has no antitumor activity in the absence of light irradiation while empty NP with light irradiation significantly inhibited the growth of tumors. DOX + Ce6 with light irradiation showed almost similar antitumor efficacy. The growth of tumor volume, especially, was mostly inhibited when mice were treated DOX-NP under light irradiation. These results indicate that DOX-NP has superior antitumor activity against breast cancer cells in vitro/in vivo. Biodistribution of DOX-NP supported the in vivo antitumor activity of DOX-NP (Figure 7b). DOX-NP was i.v. administered via the tail vein of mice to observe in vivo biodistribution (Figure 7b). Fluorescence intensity was strongest in tumor tissue while other organs revealed little or negligible fluorescence intensity, indicating that DOX-NP was efficiently delivered to tumor tissue rather than other tissues.

## 3. Discussion

The tumor microenvironment has quite different physicochemical properties compared to normal biological systems [33,34,35,36]. The tumor microenvironment (TME) has abnormalities such as acidic pH, higher redox potential, elevated metabolism and overexpression of various receptors. The oxidative stress of the tumor microenvironment, especially, has a deep relationship with cancer progression and metastasis [36]. Kuo et al. reported that ROS elevates carcinogenic protein in cancer cells and then enhances the immunosuppressive microenvironment with metastatic behaviors of cancer cells [36]. Paradoxically, the elevated redox potential of the tumor microenvironment has been used as a therapeutic target using various nanocarriers [37,38]. For example, Ayyanaar et al. reported that engineered nanocarriers revealed acidic pH and ROS sensitivity and then showed enhanced cytotoxicity against MCF-7 breast cancer cells [37]. Increased oxidative stress in the TME can be considered as a targeting issue for ROS-sensitive nanoparticles, i.e., ROS-sensitive nanoparticles can be specifically degraded under elevated oxidative stress in the TME while nanoparticles still remain in intact form. Our results also showed that empty NPs (HA(Ce6)-*b*-PEG copolymer nanoparticles) responded to H_2_O_2_ concentration and then they disintegrated (Figure 2A,B). Chen et al. also reported that thioketal-functionalized nanoparticles respond to H_2_O_2_ concentration and then accelerate the drug release rate from nanoparticles [39]. Furthermore, their nanoparticles were also degraded under oxidative stress. Nanoparticles sensitively killed cancer cells, effectively targeted tumor tissue and then effectively inhibited tumor growth. Cai et al. also reported that a dimeric cabazitaxel (CTX) prodrug is linked via the thioketal group and then assembled with Ce6 to form nanoparticles [40]. They argued that the elevated ROS level in the TME acts as a molecular trigger to cleave the TK linkers and then induces on-demand release of active CTX monomers specifically within the TME. This ROD-triggered drug delivery induced superior cellular uptake and amplified the therapeutic effect against the tumor xenograft model. In our system, moieties of Ce6, which is a hydrophobic and fluorescent dye, were attached to the HA backbone of the HA-*b*-PEG copolymer as shown in Figure S2. The HA-*b*-PEG copolymer is fully hydrophilic and dissolved in water and this copolymer cannot form self-assemblies. Since Ce6 has lipophilic properties, it was conjugated via thioketal diamine and then a Ce6-thioketal amine was attached to the carboxyl group of HA to grant hydrophobic properties against the HA-*b*-PEG copolymer (Figure 1b). Then, the HA(Ce6)-*b*-PEG copolymer (Empty NP) is able to form self-assemblies and then form nanoparticles. Since the thioketal group can be degradable under oxidative stress [39,40], nanoparticles can be disintegrated in the presence of H_2_O_2_ and, after that, empty NPs must be changed to water-soluble form, the HA-*b*-PEG copolymer. Then, this process accelerates the drug release rate as shown in Figure 2B(a,b). ROS-triggered drug release might amplify anticancer/antitumor activity and targeting potential against the tumor xenograft model (Figure 5 and Figure 7). Since glutathione (GSH) levels in the tumor microenvironment are also elevated, GSH-responsive nanocarriers can be rapidly delivered to MDA-MB-231 cells and then apoptotic protein can be upregulated by treatment of nanoparticles [38,41]. Yoon et al. reported that redox-sensitive nanoparticles were efficiently delivered DOX-resistant oral cancer cells to the intracellular region while intracellular delivery of DOX itself was significantly delayed [41]. Then, they argued that DOX-incorporated chitosan nanoparticles efficiently inhibited the viability of DOX-resistant oral cancer cells in vitro and tumor growth in vivo. Our results also revealed that intracellular delivery of DOX-NP was significantly higher than that of DOX itself against DOX-resistant cancer cells (Figure 3). Then, DOX-NP efficiently inhibited the viability of DOX-resistant MDA-MB-231 cells (Figure 5b). Multidrug resistance (MDR) is one of the major obstacles in chemotherapy using traditional anticancer agents. These problems can be overcome by designing nanocarriers to be sensitive to oxidative stress in the tumor microenvironment [41,42,43]. Our DOX-NP was designed to respond to ROS and then accelerate Ce6/DOX release (Figure 2). They responded to ROS and then disintegrated by ROS level in biological systems. Furthermore, PDT using DOX-NP inhibited the viability of DOX-resistant MDA-MB-231 cells and induced apoptotic/necrotic death (Figure 5 and Figure 6). These results were due to the higher intracellular delivery of DOX-NP against DOX-resistant MDA-MB-231 cells that efficiently produced the ROS intracellular region (Figure 4). DOX-NP treatment of cancer cells together with PDT also significantly inhibited tumor growth in an in vivo animal tumor model (Figure 7). These superiorities of DOX-NP were due to the fact that they were efficiently delivered to tumor tissues. Gong et al. also reported that the metal–organic framework (MOF) nanocarrier co-encapsulated with doxorubicin (DOX) and HIF-1α antisense oligonucleotide labeled with the Ce6 photosensitizer reverses the drug resistance of breast cancer cells and then induces death of breast cancer cells [29]. They argued that MOF nanocarriers significantly enhanced intracellular accumulation of DOX and then the combination of DOX with Ce6 initiated antitumor immunity and resulted in enhanced antitumor activity in vivo.

PDT has been believed to be a safe treatment regimen because it is composed of non-toxic components such as photosensitizers, visible light and oxygen [44]. Unlike chemotherapeutic agents, photosensitizers such as Ce6 have negligible cytotoxicity in both tumor cells and normal cells. Photosensitizers only produce ROS under field of light irradiation, i.e., light irradiation against tumor cells induces overproduction of ROS by photosensitizers, which is accumulated in the tumor tissues, and then specifically kills the tumor cells [44,45]. However, PDT has some drawbacks, which limit clinical application of novel photosensitizers [46,47]. Intrinsic properties of photosensitizers include non-specificity against tumor cells/tissues and free distribution to the whole body [48]. These non-specific deliveries of photosensitizers cause a light sensitivity/burning effect against skin [48]. Furthermore, PDT application is limited to the mucous layer of tissues or the surface of the skin because light penetration depth is normally limited. Then, PDT is difficult to apply to tumor tissues located deeper than 15 mm from the surface [49]. Nanoparticles have been considered as a solution to these problems [50,51,52]. We previously reported that water-soluble chitosan can be used to form nanocomplexes with Ce6, increase aqueous solubility and improve penetration against tumor tissues [52]. Furthermore, nanoparticles can be designed to be sensitive to the tumor microenvironment (TME). For example, tumor cells express various molecular receptors, which can be used for drug targeting [50,51,53]. The elevated redox potential of the TME can also be applied for drug targeting, i.e., nanoparticles can be designed to be sensitive to redox potential. Sun et al. reported that ROS-sensitive prodrugs are sensitively liberated anticancer drugs and photodynamic PEG-coated nano-assemblies provide a combination of chemotherapy and PDT [53]. We also provided a combinatorial strategy of chemotherapy and PDT for the treatment of DOX-resistant cancer cells, i.e., DOX-NP effectively inhibited DOX-resistant MDA-MB-231 cells and induced apoptotic death while DOX has limited anticancer activity.

## 4. Materials and Methods

### 4.1. Chemicals

HA (hyaluronic acid sodium salt, sodium hyaluronate, HA5K, Lot. No. 026570) with an average molecular weight (M*_r_*) of 4659 Da (size-exclusion chromatography coupled to a multi-angle laser light-scattering detector from the manufacturer’s data) was purchased from Lifecore Biomedical Co., Ltd. (Chaska, MN, USA). Thioketal diamine (ThdNH_2_) was purchased from Ruixi Biotech Co., Ltd. (Xi’an, China). Methoxy poly(ethylene glycol)-succinimidyl succinate (MePEG-NHS) (M.W., 5000 g/mol from manufacturer’s information) was purchased from SunBio Co., Ltd. (Seoul, Republic of Korea). Chlorin e6 (Ce6) was obtained from Frontier Sci. Co. (Logan, UT, USA). Doxorubicin HCl (DOX HCl) was purchased from LC Lab Co. (Woburn, MA, USA). Triethylamine (TEA), *N*-(3-dimethylaminopropyl)-*N′*-ethylcarbodiimide hydrochloride (EDAC), 1-hydroxybenzotriazole (HOBt), *N*-hydroxysuccinimide (NHS), triethylamine (TEA), 2′,7′-dichlorofluorescin diacetate (DCFH-DA), 3-(4,5-dimethyl2-thiazolyl)-2, 5-diphenyl-2H-tetrazolium bromide (MTT), hydrogen peroxide (H_2_O_2_) and 2,2,2-tribromoethanol (avertin) were purchased from Sigma Aldrich Chemical Co. (St. Louis, MO, USA). Dialysis tubing having a molecular weight cutoff (MWCO) size of 500~1000 g/mol, 1000 g/mol and 8000 g/mol was purchased from Spectrum Lab, Inc. (Rancho Dominguez, CA, USA). Organic solvents, such as dimethyl sulfoxide (DMSO), ethyl alcohol (EtOH), and dichloromethane (DCM), were used as extra-pure grades.

### 4.2. Synthesis of Ce6-Conjugated HA-b-PEG (HA(Ce6)-b-PEG) Copolymer

HA-*b*-PEG copolymer: HA (932 mg ≈ 0.2 mM) was dissolved in 10 mL deionized water and then 30 mL DMSO was added. Following this, excess amount of sodium cyanoborohydride was dissolved in 10 mL deionized water and added to HA solution followed with magnetic stirring for 12 h to make reductive end of HA. After that, this solution was precipitated into an excess amount of EtOH, stored in a refrigerator (4 °C) for more than 6 h to settle down, and then the precipitates were recovered with filtration. The precipitates were added to excess amount of EtOH again and then filtered. This process was repeated three times and the resultant products were dried in vacuo more than 1 day.

MePEG-NHS (500 mg, 0.1 mM) dissolved in 20 mL DMSO was mixed with excess amount of ThdNH_2_ (194.4 mg, 1 mM) and then the mix was magnetically stirred for 24 h. Following this, resultant solution was dialyzed against distilled water using dialysis tubing (MWCO: 1000 g/mol) to remove unreacted chemicals and solvents for 2 days. Distilled water was exchanged every 2~3 h intervals. Dialyzed solution was lyophilized for 2 days and then MePEG-ThdNH_2_ conjugates were obtained.

HA with reductive end (466 mg ≈ 0.1 mM) was dissolved in 5 mL deionized water and then 20 mL DMSO was added. To this solution, MePEG-ThdNH_2_ conjugates were added and then were magnetically stirred for 2 days. After that, resultant solution was added to dialysis tubing (MWCO: 8000 g/mol) and then dialyzed against distilled water for 2 days to remove unreacted chemicals or solvents. Dialyzed solution was then lyophilized for 3 days to obtain HA-*b*-PEG copolymer. To remove unreacted MePEG, lyophilizates were precipitated into 20 mL DCM, filtered, and dried in vacuo more than 1 day. The yield of HA-*b*-PEG copolymer was more than 91% (*w*/*w*). Yield (%, *w*/*w*) = [Weight of HA-*b*-PEG copolymer/(weight of HA + weight of MePEG)] × 100.

HA(Ce6)-*b*-PEG copolymer: Carboxylic acid end of Ce6 (59.7 mg, 0.1 mM) dissolved in 10 mL DMSO was activated with equivalent amount of EDAC (19.1 mg, 0.1 mM)/NHS (11.5 mg, 0.1 mM) followed with magnetic stirring for 6 h. Then, excess amount of thioketal diamine (ThdNH_2_, 194.4 mg, 1 mM) was separately dissolved in 5 mL DMSO and then further stirred for 24 h under dark conditions. After that, resulting solution was then dialyzed using dialysis tubing (MWCO size, 500~1000 g/mol) for 1 day under dark conditions with exchange of water at 1–2 h intervals followed with lyophilization for 2 days.

HA-*b*-PEG copolymer 200 mg was dissolved in 10 mL H_2_O/DMSO mixtures (3/7, *v/v*) and then mixed with EDAC (3.84 mg)/HOBt (3.1 mg). This solution was magnetically stirred for 6 h and then 15.4 mg Ce6-ThdNH_2_ conjugates were added followed with magnetic stirring for 2 days under dark conditions. After that, reactants were poured into dialysis tubing (MWCO size, 8000 g/mol) and then dialyzed against distilled water for 2 days with exchange of water at 3 h intervals. Dialyzed solution was analyzed or lyophilized for 2 days. The yield of HA(Ce6)-*b*-PEG copolymer was calculated as follows: Yield (%, *w*/*w*) = [weight of HA(Ce6)-*b*-PEG copolymer/(weight of HA-*b*-PEG copolymer + weight of Ce6-selenocystamine conjugates)] × 100. Yield of HA(Ce6)-*b*-PEG copolymer was higher than 94%.

Ce6 contents were measured as follows: HA(Ce6)-*b*-PEG copolymer (5 mg) was reconstituted into phosphate-buffered saline (PBS, 0.01 M, pH 7.4) with 10 mM H_2_O_2_ and then incubated at 37 °C under shaking (100 rpm) for 3 h. This solution was diluted with DMSO at least 10 times and then Ce6 concentration was measured using an ultraviolet–visible spectrophotometer (UV-1601 UV-VIS spectrophotometer, Shimadzu, Kyoto, Japan) at 664 nm. For comparison, similar contents of HA-*b*-PEG copolymer were dissolved in PBS in the presence of H_2_O_2_ and then diluted with DMSO. Ce6 alone dissolved in DMSO was also added to PBS in the presence of H_2_O_2_, incubated at 37 °C under shaking (100 rpm) for 3 h, and then diluted with DMSO for comparison.

Ce6 contents in the conjugates: Ce6 content (*w*/*w*, %) = (weight of Ce6/total weight of conjugates) × 100.

Theoretical contents of Ce6 in HA(Ce6)-*b*-PEG copolymer were calculated as 5.55% (*w*/*w*). Experimental contents measured by above equation were higher than 5.4% (*w*/*w*).

### 4.3. Fabrication of DOX-Incorporated Nanoparticles

HA(Ce6)-*b*-PEG copolymer (50 mg) was reconstituted into 5 mL distilled water and then 3 mL DMSO was added. DOX (5 mg; As a DOX HCl, 5.35 mg (9.2 mM)) with equivalent mole of TEA was separately dissolved in 2 mL DMSO. DOX solution was added to solution of HA(Ce6)-*b*-PEG copolymer, magnetically stirred for 30 min, and then poured into dialysis tubing (MWCO size, 8000 g/mol). This was dialyzed against distilled water for 1 day with exchange of water at 1–2 h intervals. Resulting solution was used for analysis or lyophilized for 2 days.

To calculate DOX content in the nanoparticles, volume of dialyzed solution was adjusted to 20 mL and 1 mL of this solution was diluted with DMSO ten times and then absorbance of this solution was measured using UV spectrophotometer at 479 nm. DOX content: DOX content (*w*/*w*) = (DOX weight in the nanoparticles/nanoparticle weight) × 100.

### 4.4. Characterization of HA(Ce6)-b-PEG Copolymer and DOX-NP

Synthesized polymers were analyzed with ^1^H NMR spectra (500 mHz Agilent ProPulse NMR system, Agilent Tech. Inc., Santa Clara, CA, USA). Each chemical or polymer was dissolved in DMSO or D_2_O/DMSO mixtures (3/7, *v*/*v*). Deuterated solvent such as DMSO or D_2_O was used for NMR spectra.

Particle size analysis was carried out with Nano-Zetasizer (Nano-ZS, Malvern, Worcestershire, UK). Nanoparticles in deionized water were used to analyze particle size distribution. Measurement was repeated three times and then expressed as average diameter ± standard deviation (SD).

To study the effect of ROS, NP and DOX-NP (5 mg) was reconstituted in 5 mL deionized water or phosphate-buffered saline (PBS, 0.01 M, pH 7.4) in the presence of H_2_O_2_. Then they were incubated for more than 3 h at 37 °C. Then, these were used to measure particle size and to observe morphology.

### 4.5. Drug Release Study

Ce release study was performed as follows: nanoparticles of HA-*b*-PEG copolymer (NP) (5 mg) were reconstituted into 5 mL deionized water and then introduced into dialysis tube (MWCO, 8000 g/mol). Dialysis bag was introduced into 50 mL conical tube with 45 mL PBS in both conditions of with and without H_2_O_2_. Following this, these were incubated in the shaking incubator (100 rpm) at 37 °C. To check released Ce6 from nanoparticles, whole PBS was taken and replaced with fresh PBS to prevent saturation of drug. Released Ce6 was measured with UV-VIS spectrophotometer at 664 nm. All experiments were carried out in dark condition, and the results were expressed as average ± standard deviation (SD) from three different experiments.

DOX release study was performed as follows: DOX-NP (5 mg) reconstituted into 5 mL of deionized water was introduced into a dialysis tube (MWCO, 8000 g/mol). Dialysis bag was introduced into 50 mL conical tube with 45 mL phosphate-buffered saline (PBS, 0.01 M, pH 7.4) in both conditions of them being with and without H_2_O_2_. Following this, these were incubated in the shaking incubator (100 rpm) at 37 °C. To check released DOX concentration, whole PBS was taken and replaced with fresh PBS to prevent saturation of drug. Released DOX was measured with UV-VIS spectrophotometer at 479 nm. This process was repeated three times and then expressed as average ± standard deviation (SD).

### 4.6. Cell Culture Study

For cell culture study, MDA-MB-231 human breast cancer cells, purchased from the Korean Cell Line Bank (Seoul, Republic of Korea), were maintained with RPMI 1640 medium (supplemented with 1% antibiotics and 10% fetal bovine serum) under 5% CO_2_ and 37 °C.

DOX-resistant MDA MB cells were prepared as follows: DOX (0.001 μg/mL) were exposed to cells for 12 h and then replaced with fresh media followed with culture at 37 °C (5% CO_2_) for 2~3 days. After that, cells were exposed to DOX (0.001 μg/mL) once more for 12 h. DOX concentration was gradually increased from 0.001 μg/mL to 0. 1 μg/mL for three months (DOX-resistant MDA-MB-231 cells).

For chemotherapeutic treatment, DOX-resistant MDA-MB-231 cells (1 × 10^4^ cells/well) seeded in 96 wells were incubated at 5% CO_2_ (37 °C) overnight. After that, DOX, NP, DOX + Ce6 or DOX-NP were treated to cells. For DOX or DOX + Ce6 treatment, DOX or DOX + Ce6 dissolved in DMSO was diluted to appropriate concentration with cell culture media at least 100 times. For NP or DOX-NP treatment, they were sterilized with a syringe filter (0.8 µm) and then diluted with cell culture media. These cells were incubated for 1 d (5% CO_2_) at 37 °C. After that, cell viability was analyzed with MTT cell viability test. MTT solution (30 μL, 5 mg/mL in PBS) was added to the cells and then further incubated 3 h at (5% CO_2_) at 37 °C. Following this, the supernatants were removed and 100 µL of DMSO was added. Then, absorbance of these samples was measured at 570 nm using a microplate reader (Infinite M200 pro multimode microplate reader, Tecan Trading AG Inc., Männedorf, Switzerland). All results of the cell cytotoxicity study were expressed as average ± SD from 8 wells.

For PDT study, DOX-resistant MDA-MB-231 cells (1 × 10^4^ cells/well) seeded in 96 wells were treated with Ce6 or NP. For Ce6 treatment, Ce6 dissolved in DMSO was diluted with media 100 times and then treated to cells. NP was sterilized with a syringe filter (0.8 µm) and then diluted with cell culture media. Cells were cultured in CO_2_ incubator (5% CO_2_) at 37 °C for 2 h. Cells were irradiated with an expanded homogenous beam (SH Systems, Gwangju, Republic of Korea) at 664 nm. The dose of light was 2.0 J/cm^2^ (Light dose was determined by measurement with a photo radiometer (Delta Ohm, Padua, Italy)). Cells were further incubated for 22 h in CO_2_ (37 °C) and then cell viability was measured with MTT assay.

For apoptosis/necrosis analysis of DOX-resistant MDA-MB-231 cells, 3 × 10^5^ cells seeded in dish were treated with DOX or DOX-NP for 24 h in the 5% CO_2_ incubator at 37 °C. Following this, cells were harvested by centrifugation, washed with PBS and then resuspended in binding buffer (10 mM HEPES, pH 7.4, 150 mM NaCl, 5 mM KCl, 1 mM MgCl_2_, 1.8 mM CaCl_2_). After that, cells were treated with FITC-annexin V (1 g/mL, sc-4252 FITC, Santa Cruz Biotech., Inc., Dallas, TX, USA) and propidium iodide (PI, 10 g/mL) for 20 min to stain apoptotic cells and necrotic cells, respectively. Apoptosis/necrosis of cancer cells was analyzed using a flow cytometer (FACScan flow cytometer, Becton Dickinson Biosciences, San Jose, CA, USA) at 488 nm (FITC-annexin) and 575 nm (PI). All procedures were performed in the dark.

For fluorescence observation of cells, cells (2 × 10^5^) were seeded on to the cover glass placed in six-well plates and then cultured overnight. Cells were treated with DOX or DOX-NP for 2 h. Following this, cells were washed with PBS and then fixed with 4% paraformaldehyde (PFA) solution for 15 m and then immobilized with immobilization solution (Immunomount, Thermo Electron Co., Pittsburgh, PA, USA). Cells were observed with a fluorescence microscope (Emission bandwidth: 600–660 nm) (Eclipes 80i; Nikon, Tokyo, Japan).

For analysis of ROS generation, cells (2 × 10^4^ cells/well) in 96-well plate were exposed to Ce6 or NP in serum-free/phenol-red-free media with DCFH-DA (final concentration: 20 µM) for 2 h. Following this, cells were washed with PBS twice and then replaced with 100 µL of fresh serum-free/phenol red-free RPMI media followed by irradiation of cells at 664 nm (2.0 J/cm^2^). The generated intracellular ROS was measured at an excitation wavelength of 485 nm and emission wavelength of 535 nm using microplate reader.

### 4.7. In Vivo Animal Tumor Study Using Tumor Xenograft Model

Male BALB/c nude mice (20–25 g, 4–5 weeks old) were purchased from Orient (Seongnam, Republic of Korea) for antitumor activity study using DOX-NP. All mice were freely fed with supported water until use.

DOX-resistant MDA-MB-231 cells (1 × 10^7^ cells) were subcutaneously administered to the backs of the mice. When the solid tumor was 4–5 mm, its largest diameter, drug or nanoparticles were i.v. administered. For control group, PBS (pH7.4, 0.01 M, 100 µL) was i.v. administered via tail vein of mice. Drug treatment group was randomized as follows: Empty NP; DOX; DOX + Ce6 with light irradiation; Empty NP with light irradiation; DOX NP with light irradiation. For DOX or DOX + Ce6 injection, DOX or DOX + Ce6 was dissolved in ethanol/Cremophor EL and diluted at least 10 times with PBS (pH 7.4, 0.01 M). Empty NP or DOX-NP were reconstituted in deionized water and then sterilized with a 1.2 µm syringe filter. For empty NP, similar amount of empty NP compared to DOX NP was weighted and distributed in PBS. DOX dose at DOX, DOX + Ce6 and DOX-NP treatment was adjusted to be 10 mg/kg. Ce6 dose at NP or DOX-NP was adjusted to be 6 mg/kg since Ce and DOX contents in DOX NP were 4.9% (*w*/*w*) and 8.9% (*w*/*w*).

These were administered intravenously via tail vein of mice. For each treatment group, five mice were used and injection volume was 100~200 µL. Mice were freely fed food and water (3~4 mice/cage). The period of drug injection was determined as Day 0. After 3 d and 5 d, mice were anesthetized and then irradiated at 664 nm using the expanded homogenous beam (SH Systems, Gwangju, Republic of Korea) with 5.0 J/cm^2^.

The growth of tumor volume was measured at 5 d intervals and then calculated. V (Tumor volume) = [a × (b)^2^]/2. a = largest diameter; b = smallest diameter. Results from animal experiments were expressed as average ± SD from five mice. To minimize confounders, measurement of tumor size was repeated by three researchers and then the average was calculated.

Whole body and organ optical imaging was carried out as follows: DOX-NP (10 mg/kg as a DOX) was i.v. administered via tail vein of mice having tumor xenograft (Injection volume: 200 µL). After 24 h, mice were anesthetized with avertin to observe whole body imaging using a Maestro^TM^ small animal imaging instrument (Cambridge Research and Instruments, Inc., Woburn, MA, USA) in the dark. Mice were sacrificed for fluorescence observation of each organ.

### 4.8. Statistical Analysis

The significance of statistical analysis was performed with Student’s *t* test using the SigmaPlot^®^ program (v. 11.0, Systat Software, Inc., Palo Alto, CA, USA). In statistical analysis, the minimum value of significance was estimated as *p* < 0.01.

## 5. Conclusions

The HA(Ce6)-*b*-PEG copolymer was synthesized for ROS-sensitive delivery of Ce6 and DOX. DOX-NP has a smaller diameter than 200 nm and has a spherical morphology. They disintegrated in an ROS-sensitive manner and then the Ce6 or DOX release rate accelerated. Intracellular delivery of DOX-NP was significantly higher than that of DOX itself and revealed superior anticancer activity against DOX-resistant MDA-MB-231 cells than that of DOX itself. Furthermore, DOX-NP greatly inhibited the viability of DOX-resistant MDA-MB-231 cells under light irradiation, indicating that PDT of DOX-resistant MDA-MB-231 cells using DOX-NP induced death of cancer cells. Also, DOX-NP more efficiently induced apoptosis/necrosis than DOX itself. In DOX-resistant MDA-MB-231 cell-bearing mice, DOX-NP was efficiently delivered to tumor tissue rather than normal organs and then greatly inhibited the growth of tumors under light irradiation. We suggest that DOX-NP is a superior candidate for an anticancer therapeutic strategy.

## Figures and Tables

**Table 1:** Characterization of DOX NP.

	DOX Contents (%, *w*/*w*)	Loading Efficiency (%, *w*/*w*)	Particle Size (nm) ^a^	Polydispersity Index	Zeta Potential (mV)
Empty NP ^b^	-	-	98.0 ± 5.8	0.313	−10.68
DOX NP ^b^	8.9 ^c^	97.8	134.6 ± 6.4	0.289	−9.67

^a^ Average particle sizes were average ± SD from three measurements. ^b^ Empty NP means nanoparticles of HA(Ce6)-*b*-PEG copolymer. DOX NP means DOX-incorporated nanoparticles of HA(Ce6)-b-PEG copolymer. ^c^ When DOX contents in the DOX NP were 8.9% (*w*/*w*), Ce6 content in the empty NP and DOX NP was calculated as 5.4% (*w*/*w*) and 4.9% (*w*/*w*).

**Table 2:** IC_50_ value of DOX, DOX NP, Ce6 and empty NP against DOX-resistant MDA-MB-231 cells.

	Light Irradiation	IC_50_ (µg/mL) ^a^
DOX	-	>10
DOX NP ^b^	-	4.6
DOX	+	>10
DOX NP ^b^	+	0.44
Ce6	-	>5
Empty NP ^b^	-	>5
Ce6	+	>5
Empty NP ^b^	+	0.64

^a^ IC_50_ values were estimated from Figure 5. ^b^ IC_50_ values were estimated from Figure 5a,b. When DOX concentration in the DOX NP was 0.44 µg/mL, Ce6 contents in the DOX NP were calculated as 0.24 µg/mL. At 0.2 µg/mL Ce6 in empty NP, cell viability of empty NP under light irradiation was 64.05%.

## Data Availability

The original contributions presented in this study are included in the article/Supplementary Material. Further inquiries can be directed to the corresponding authors.

## Supplementary Materials

The following supporting information can be downloaded at: https://www.mdpi.com/article/10.3390/ijms27156993/s1.
