# Supplementary material for: Doxorubicin-Incorporated Nanoparticles Composed of Ce6-Conjugated Hyaluronic Acid-b-poly(ethylene glycol) Copolymer for Overcoming Doxorubicin Resistance of Breast Cancer Cells
Source: Int J Mol Sci. 2026 Aug 4;27(15):6993. doi: 10.3390/ijms27156993 (PMC13466280; doi:10.3390/ijms27156993)
Supplement: Supplementary file 1 [file ijms-27-06993-s001.zip › ijms-4335105-supplementary.pdf]

Supplementary information

Experimental

Fourier transform infrared (FT-IR) spectroscopy

FT-IR spectroscopy was employed to analyze the chemical structures of HA(Ce6)-b-PEG copolymer and each component (Shimadzu, FT-IR 8000, Japan). Powders of each component were compressed using a force of 5 t in a hydraulic press and prepared KBr pellets and then recorded in the 4000 – 400 cm^{-1} range at room temperature.

Intracellular Ce6 uptake

The cancer cells (2×10^4 cells) were seeded in a 96-well plate and then exposed to Ce6 or HA(Ce6)-b-PEG copolymer for 2 h. For the Ce6 treatment, it was dissolved in DMSO and then diluted with serum-free media more than 100 times. The HA(Ce6)-b-PEG copolymer was also diluted with serum-free media. Each treatment was incubated for 2 h in a 5%CO_2 incubator at 37 ◦C. After that, the cells were washed with PBS twice and solubilized in 50 µL of lysis buffer (GenDEPOT, Barker, TX, USA). Then, the Ce6 uptake ratio was measured with an Infinite M200 pro microplate reader (Tecan) (excitation wavelength: 407 nm; emission wavelength: 664 nm).

Protein concentration was measured using Pierce BCA protein assay kit (Thermo Fisher Scientific Co., Waltham, USA).

Intracellular ROS generation

For intracellular ROS level, cells (2×10^4 cells) were seeded in a 96-well plate and then exposed to Ce6, DOX, DOX+Ce6, HA(Ce6)-b-PEG copolymer or DOX-NP for 2 h as described above. To measure the generation of intracellular ROS, a DCFH-DA assay was employed. The cells were exposed to each chemicals in phenol red free RPMI media in the presence of DCFH-DA (final concentration: 20 µM) for 2 h at 37 ◦C. Following this, the cells were washed with PBS twice, replaced with 100 µL fresh phenol red free RPMI media and then irradiated at 664 nm (2.0 J/cm2). Intracellular ROS generation was estimated with a microplate reader (Infinite M200 pro microplate reader (Tecan); excitation wavelength: 485

nm; emission wavelength: 535 nm).

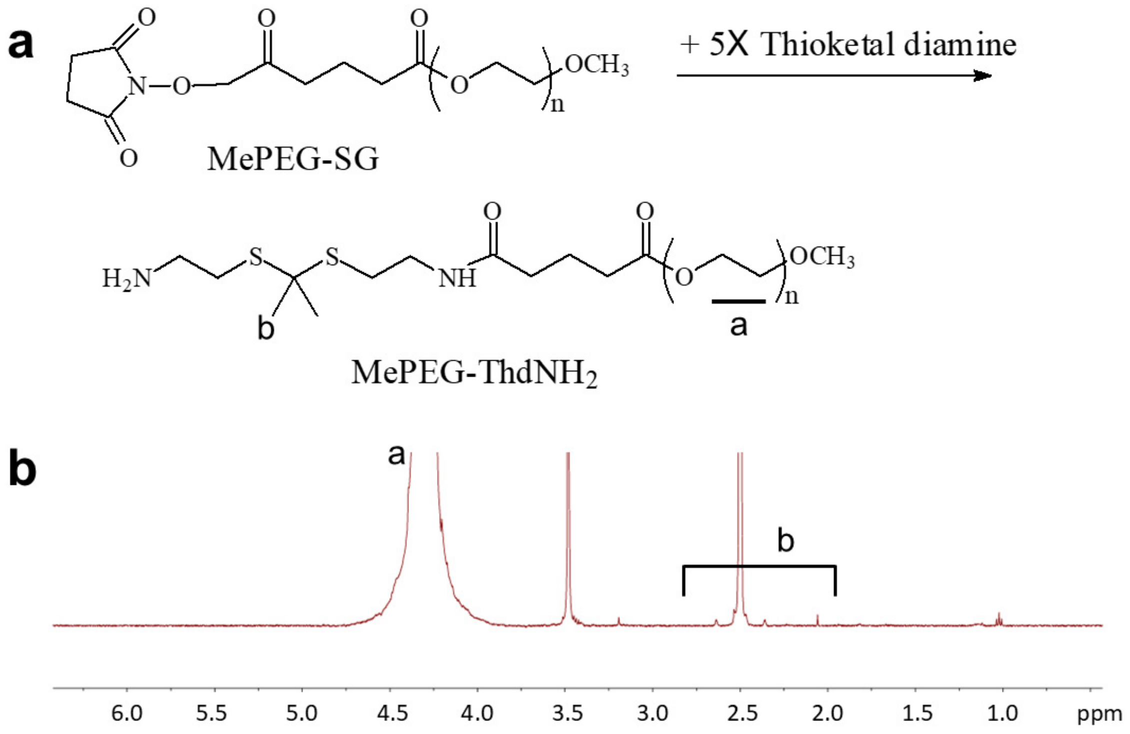

Figure S1. Synthesis scheme (a) and ^{1}H NMR spectra of MePEG-ThdNH$_2$ conjugates.

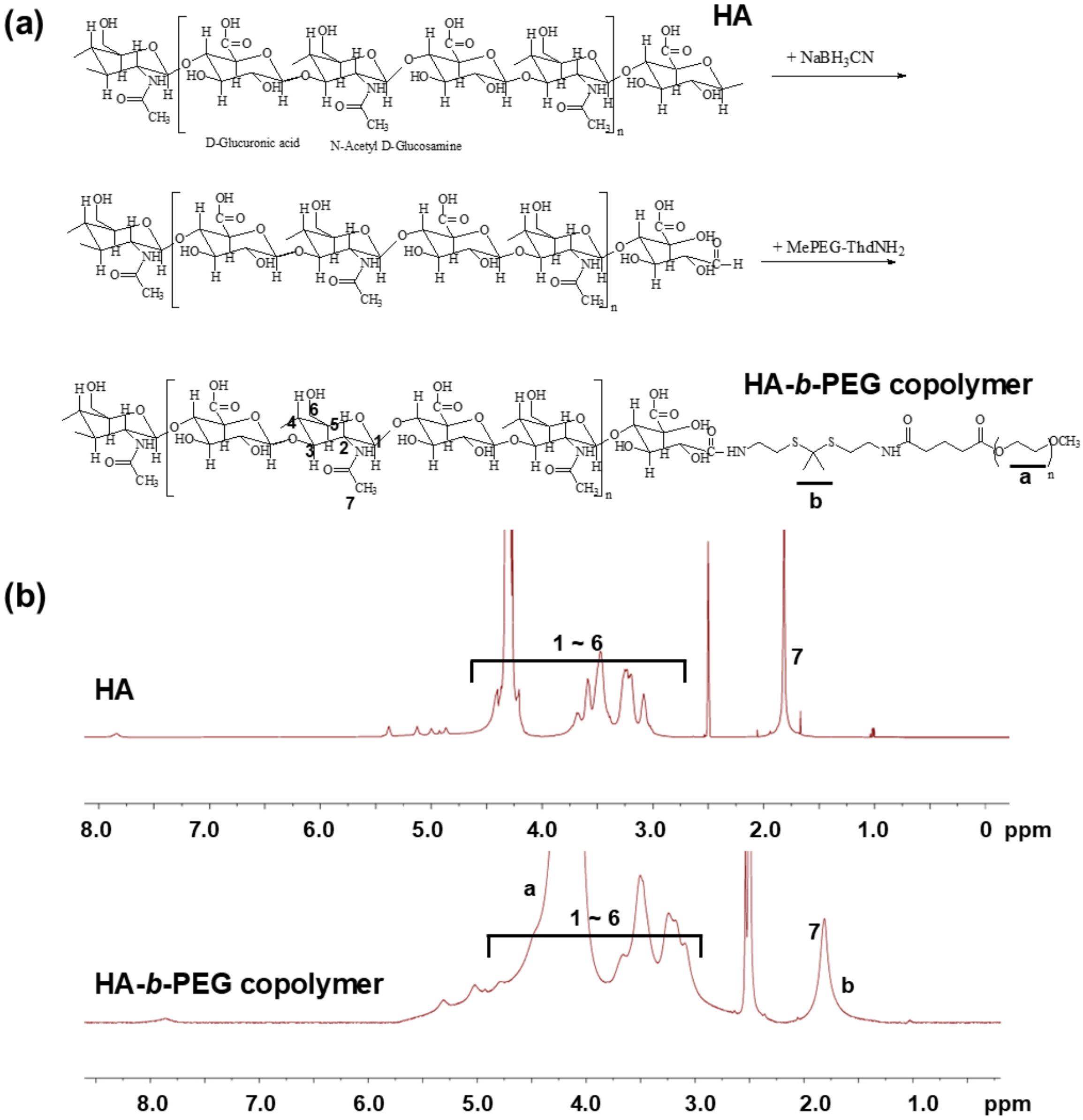

Figure S2. Synthesis scheme (a) and ^{1}H NMR spectra of HA-*b*-PEG copolymer.

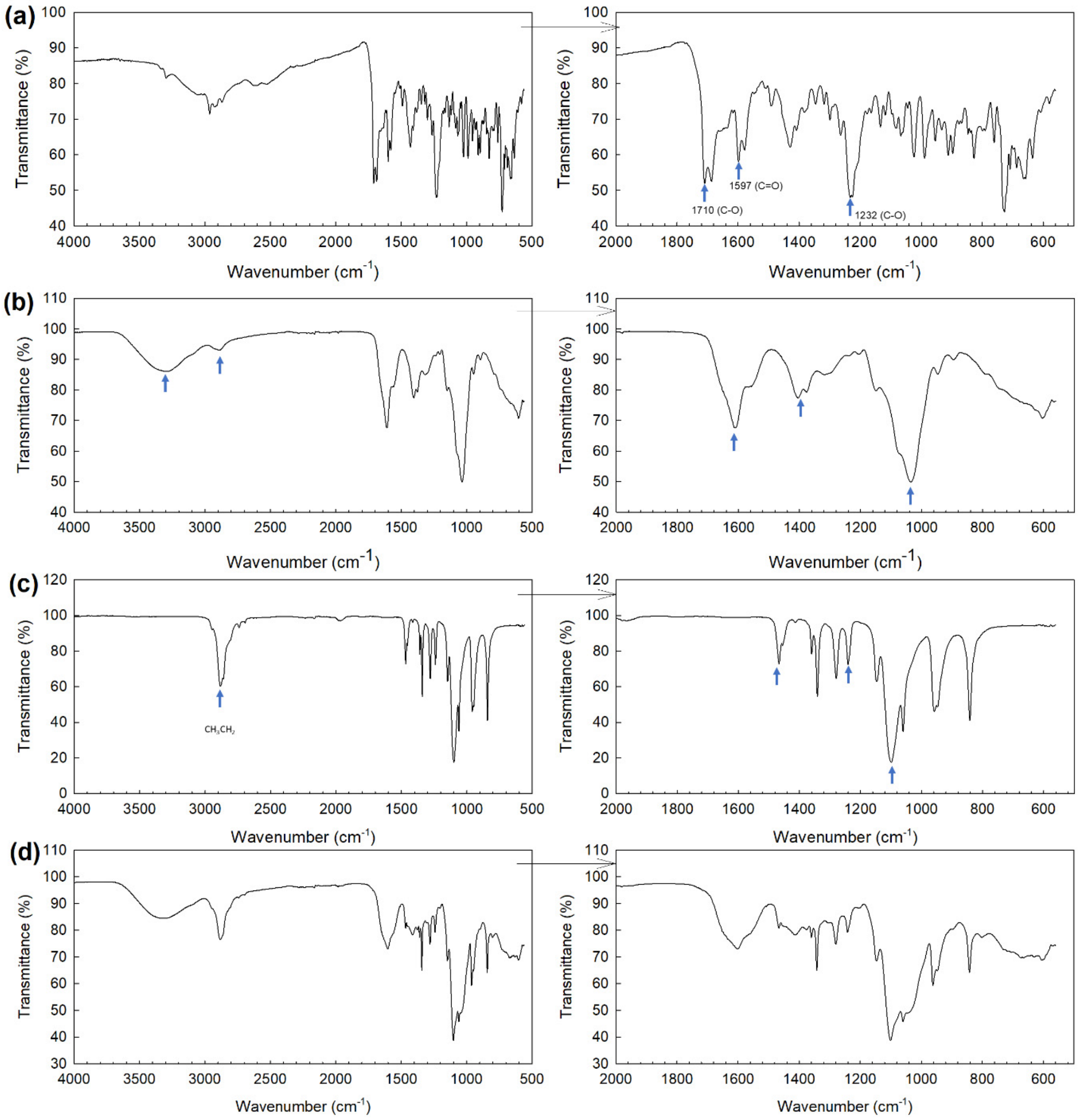

Figure S3. FT-IR spectra of Ce6 (a), HA(b), PEG (c) and HA(Ce6)-*b*-PEG copolymer (d).

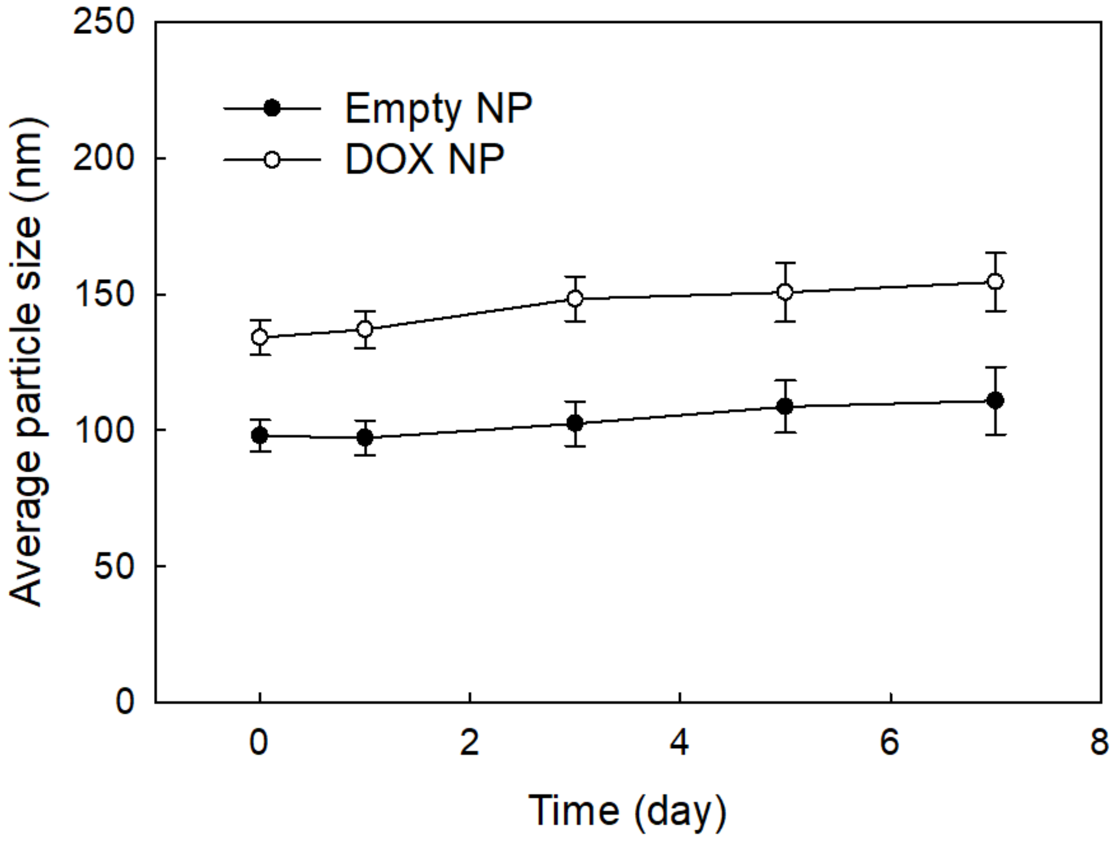

Figure S4. Time course of colloidal stability of empty NP (HA(Ce6)-*b*-PEG copolymer nanoparticles) and DOX NP (DOX-incorporated HA(Ce6)-*b*-PEG copolymer nanoparticles). Empty NP and DOX NP were stored in 4°C and then measured with zeta-sizer.

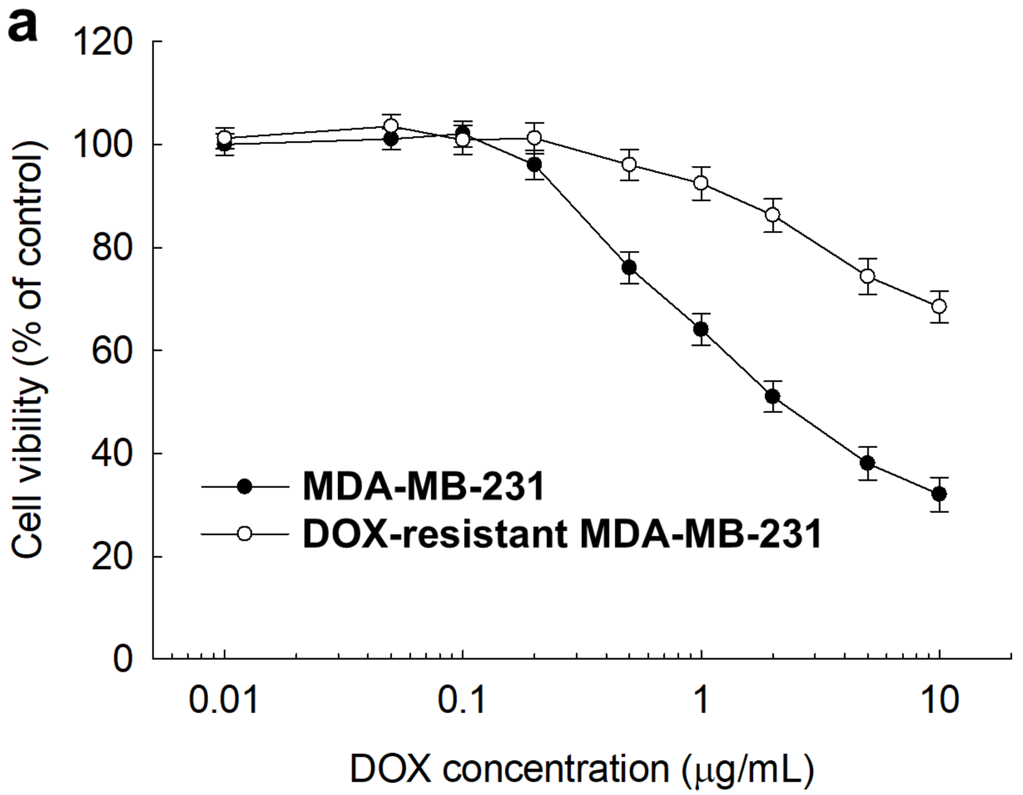

b

	IC_{50} (μg/mL)
MDA-MB-231	2.1
DOX-resistant MDA-MB-231	> 10

Figure S5. (a) The effect of DOX on the viability of MDA-MB-231 cells and DOX-resistant MDA-MB-231 cells. (b) IC_{50} value of cancer cells against DOX treatment. IC_{50} value was estimated from Figure S4(a).

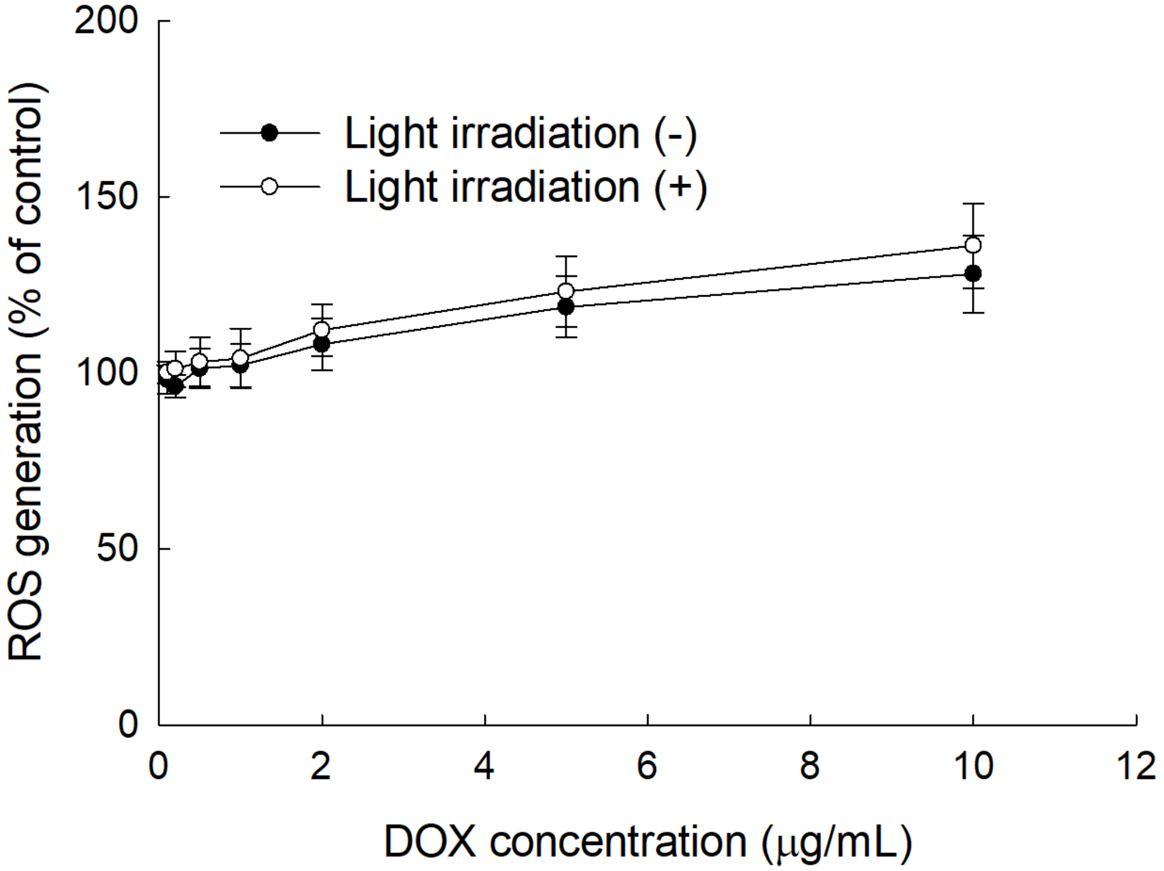

Figure S6. The effect of free DOX and light irradiation on the generation of intracellular ROS. Cells were irradiated at 664 nm (2 J/cm2). All values are average ± S.D. from results of a single independent experiment with eight replicates
